# The Effects of Exercise on White and Brown Adipose Tissue Cellularity, Metabolic Activity and Remodeling

**DOI:** 10.3389/fphys.2021.772894

**Published:** 2021-11-02

**Authors:** Jacob D. Garritson, Sihem Boudina

**Affiliations:** Department of Nutrition and Integrative Physiology, College of Health, University of Utah, Salt Lake City, UT, United States

**Keywords:** exercise, training, white adipose tissue, brown adipose tissue, inflammation, progenitors

## Abstract

Emerging evidence suggests a significant functional role of adipose tissue in maintaining whole-body metabolic health. It is well established that obesity leads to compositional and morphological changes in adipose tissue that can contribute to the development of cardiometabolic disorders. Thus, the function and size of adipocytes as well as perfusion and inflammation can significantly impact health outcomes independent of body mass index. Lifestyle interventions such as exercise can improve metabolic homeostasis and reduce the risk for developing cardiometabolic disorders. Adipose tissue displays remarkable plasticity in response to external stimuli such as dietary intervention and exercise. Here we review systemic and local effects of exercise that modulate white and brown adipose tissue cellularity, metabolic function and remodeling in humans and animals.

## Introduction

Obesity continues to be a growing public health concern and has detrimental economic impacts on healthcare systems worldwide. In general, an increase in body mass index (BMI) is associated with increased rates of development and mortality from cardiovascular disease and various cancers (Berrington De Gonzalez et al., 2010). While this relationship is well established, emerging evidence suggests that the location, composition and function of adipose depots may play a role in overall metabolic health independent of BMI. For example, it is believed that *de novo* recruitment and differentiation of preadipocytes following chronic overnutrition results in smaller, metabolically healthy adipocytes when compared to hypertrophic growth of existing adipocytes. Moreover, smaller adipocytes have been shown to be protective against metabolic decline (Lundgren et al., 2007; McLaughlin et al., 2007) and adipocyte size may be used as a predictor for the development of insulin resistance in obese individuals (Lönn et al., 2010; Yang et al., 2012; Verboven et al., 2018). Adipose tissue has traditionally been thought of simply as an energy storage depot, however, we are beginning to appreciate the exceptional plasticity of adipose tissue in response to environmental stimuli and the functional role that it plays in metabolic homeostasis.

Adipose tissue is a complex organ composed of different cells and niches that control key processes including adipogenesis, adipokine secretion and inflammatory responses. While mature adipocytes are the main functional cells regulating lipid uptake and release, they only constitute a fraction (about 20% in humans) of the cells contained in this tissue (Lee et al., 2013). Other cells such as endothelial cells, immune cells, adipose progenitor cells (APCs), preadipocytes, fibroblasts and neural cells can be found with varying proportions in the stromal vascular fraction (SVF) in each fat depot. Here we will examine the effect of exercise on white and brown adipose tissue cellularity, metabolic function and remodeling in the context of obesity. We will contrast findings in humans and rodents and discuss areas of future investigation.

## Effects of Exercise on Adipose Tissue Cellularity

Effects of exercise on adipose tissue cellularity in rats have been observed as early as the 1970’s. This early work highlights the potential of exercise to favorably modulate adipose tissue cellularity, with exercising groups consistently presenting with smaller adipocytes in white adipose tissue (Oscai et al., 1972; Askew et al., 1975; Askew and Hecker, 1976). These effects appear to be consistent across subcutaneous (SUB) and visceral (VIS) fat depots. For example, treadmill exercise in weight-reduced Wistar rats resulted in smaller adipocytes in both VIS and SUB fat (Higgins et al., 2011). Additionally, exercise in rats during weight regain increased cell number and decreased cell size in both SUB and VIS fat pads (Giles et al., 2016). While exercise appears to positively affect adipose tissue cellularity in both VIS and SUB fat pads, these effects may be mediated through different mechanisms. Sertie et al. (2019) found that cessation of exercise resulted in increased size of SUB and VIS fat pads. In VIS fat this increase was due primarily to fat cell hypertrophy, whereas, in SUB fat they observed both fat cell hypertrophy, hyperplasia, and reduced apoptosis (Sertie et al., 2019). Stimulation of lipogenesis and disruption of angiogenesis may contribute to the increase in size and dysfunction of adipocytes with obesity. Exercise is known to stimulate lipolysis (Woo and Kang, 2016), decrease lipogenic gene expression in adipose tissue (Giles et al., 2016), and promote angiogenesis through an increased expression of murine double minute-2 (Loustau et al., 2020). These three mechanisms may explain how exercise promotes smaller, metabolically healthy adipocytes by limiting hypertrophic growth and hypoxia in adipose tissue.

In comparison to the work in animal models described above, evidence for an effect of exercise on adipose tissue cellularity in humans is lacking. This is likely due to limitations in obtaining adipose tissue biopsies, especially in VIS fat pads. Additionally, investigators are finding that the physiology of adipose depots in mice may not directly translate to human adipose tissue (Severinsen et al., 2020). In comparison to the consistent observations in animal models described above, a recent study found that there was no effect of exercise on SUB adipocyte morphology in obese men (Stinkens et al., 2018). While the effects of exercise on adipose tissue cellularity have not been studied extensively in humans, it is well established that structured exercise is effective in reducing fat mass (Sabag et al., 2017; Wedell-Neergaard et al., 2019). It is less clear, however, if similar changes in lipolysis, lipogenesis, and angiogenesis occur in human fat tissue. For example, one study found that adipose tissue triglyceride lipase activity was increased in lean and obese individuals with exercise (Petridou et al., 2017), while others have found no effect of exercise intervention on adipose tissue lipolysis (Stinkens et al., 2018). It should be noted that exercise may only acutely affect WAT lipolysis. Petridou et al. (2017) observed increased lipolysis in lean and obese men for 10–30 min following moderate intensity cycling, which returned to baseline 30 min post-exercise. Conversely, Stinkens et al. (2018) found no change in WAT lipolysis when samples were measured 72 h after the final exercise bout. Additionally, one study found that there was no change in angiogenic genes with weight loss and exercise in human adipose tissue (Cullberg et al., 2013). Evidence in animal models strongly argues that exercise promotes favorable WAT morphology, but evidence for this in humans is lacking. The discrepancy could be caused by heterogeneity of the subjects, their metabolic status and differences in exercise interventions.

Adipose tissue expands primarily through hypertrophic growth of existing adipocytes or *de novo* recruitment and differentiation (adipogenesis) of APCs. Recruitment and differentiation of preadipocytes is believed to be protective against metabolic decline by limiting cell hypertrophy, hypoxia, and adverse tissue remodeling. It has recently been demonstrated that functionally distinct populations of APCs exist in adipose tissue of mice and humans (Marcelin et al., 2017; Hepler et al., 2018; Buffolo et al., 2019; Raajendiran et al., 2019). These distinct populations are identified based on the expression level of the surface markers CD34 and CD9, respectively, with pro-adipogenic APCs described as CD34^low^ and CD9^low^ and anti-adipogenic/pro-fibrotic APCs described as CD34^hi^ and CD9^hi^. It has been demonstrated that the population of pro-fibrotic and pro-inflammatory APCs increases with obesity and promote fibrosis, inflammation, and exacerbate metabolic dysfunction (Marcelin et al., 2017; Hepler et al., 2018; Buffolo et al., 2019). Considering the positive effects of exercise on metabolic health and adipose tissue cellularity, exercise may have the potential to positively regulate APC function. Information on this topic is limited, however, a recent study found that acute exercise in obese adults resulted in a decrease in pro-fibrotic CD34^hi^ APCs and no change in CD34^low^ APCs in abdominal SUB fat in humans (Ludzki et al., 2020). This suggests that exercise may positively regulate the APC pool by limiting the amount of anti-adipogenic, pro-fibrotic APCs. Additional studies are needed to examine whether this effect is limited to SUB fat or can also occur in VIS fat considering the differences in APC populations between these depots.

In addition to modulating white adipose tissue (WAT), treadmill exercise in high fat diet (HFD) mice resulted in an increase in preadipocytes in brown adipose tissue (BAT), with increased adipogenic capacity *in vitro* and increased expression of UCP1 (Xu et al., 2011). Further, a recent study found that exercise-induced secreted factors from skeletal muscle may alter APC function (Zeve et al., 2016). The authors identified R-spondin 3 (Rspo3) from skeletal muscle of trained mice as a potential mechanism that decreases differentiation of white preadipocytes. The decrease in differentiation of APCs with exercise may seem counter-intuitive, however, this could be explained by improved health of existing adipocytes and reduced apoptosis and adipocyte turnover. More work is needed to uncover the influence of exercise on APCs and adipogenesis, however, this early work suggests that exercise has the potential to positively regulate the APC pool. What remains to be determined is if exercise modulates APC proliferation and differentiation in a depot-specific manner.

## Remodeling of Adipose Tissue With Exercise

**FIGURE 1:**
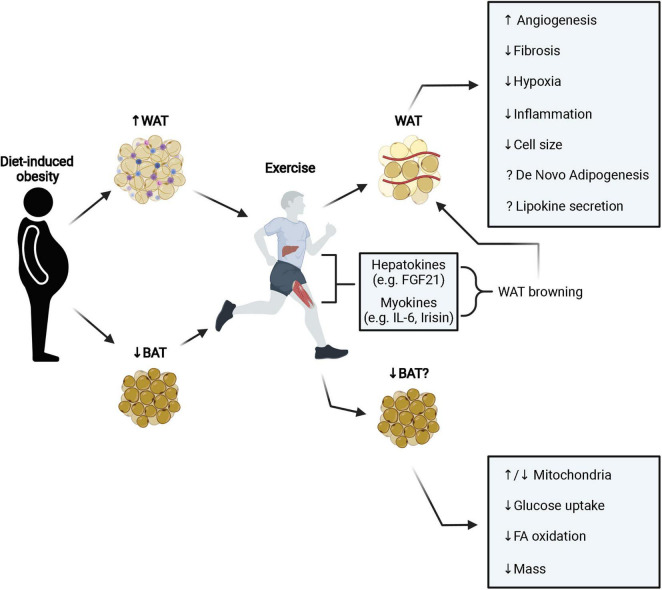
Diet induced obesity leads to pathological expansion and remodeling of WAT resulting in fibrosis, inflammation, and hypoxia. Exercise stimulates browning of WAT (primarily in mice) through the secretion of various hepatokines and myokines that improves metabolic function. Additionally, exercise stimulates angiogenesis, reduces fibrosis, hypoxia, inflammation, and cell size. It is still unclear how exercise may modulate the adipose progenitor niche and influence *de novo* adipogenesis and conflicting results have been observed in terms of lipokine secretion (e.g., adiponectin and leptin). In BAT, obesity decreases tissue mass and metabolic function. Interestingly, exercise appears to also decrease BAT mass and metabolic activity, however, this does not prevent improvements to systemic metabolic health with exercise. White adipose tissue; WAT, brown adipose tissue; BAT, fatty acid; FA, fibroblast growth factor 21; FGF21, interleukin-6; IL-6.

There are three primary types of adipose tissue that have been very well characterized: white adipose tissue (WAT), brown adipose tissue (BAT) and beige adipose tissue. WAT has long been thought of as an energy storage depot with the primary function of storing excess lipids. It is now well established that WAT functions as an endocrine organ, secreting various adipokines that affect whole body metabolic homeostasis and normal adipose tissue function is disrupted with obesity (Trayhurn and Beattie, 2001). BAT is important for regulation of body temperature by generating heat through non-shivering thermogenesis. This type of adipose tissue is characterized by its multilocular morphology, high density of mitochondria, and increased expression of uncoupling protein 1 (UCP1). Beige adipose tissue is inducible and is recruited in response to beta adrenergic stimulation. Diet-induced obesity reduces the relative amount of BAT and aerobic exercise has been shown to reverse this effect and improve metabolic health (Fu et al., 2021). We are gaining a better appreciation of the complexity of adipose tissue physiology and the external factors that can affect it, such as exercise. Adipose tissue possesses a tremendous flexibility as it continuously remodels by changing its mass and its composition in response to internal and external stimuli. Here, we will discuss the effect of exercise on white and brown adipose tissue remodeling, with a special focus on angiogenesis, fibrosis and immune infiltration. These effects have been summarized in Figure 1.

### Angiogenic Remodeling

It has been consistently reported that diet-induced obesity results in a significant decrease in angiogenesis and capillary density in white adipose tissue (Kolahdouzi et al., 2019; Loustau et al., 2020). This angiostatic effect results in an increase in hypoxia in adipose tissue that is linked to increased inflammation and insulin resistance (Ye, 2009). There has been early work in humans that observed an acute increase in circulating endothelial progenitor cells with exercise (Rehman et al., 2004; Walther et al., 2009). While this does not directly show an increase in angiogenesis in adipose tissue with exercise, these observations suggest that repeated bouts of acute exercise may increase the potential for angiogenesis and vascular repair (Figure 1). The effect of exercise on adipose angiogenesis appears to depend on insulin sensitivity in human. Thus, Walton et al. (2015) showed that aerobic exercise training (3 days/week for 12 weeks) failed to increase vessel density in SUB of insulin resistant subjects whereas insulin sensitive subjects showed an increase. It is worth noting that this training protocol did not decrease body weight or improve insulin sensitivity, which may have influenced the pro-angiogenic effect of exercise in this tissue.

In animal models, one study found that exercise training in obese rats resulted in improved vessel density in both SUB and VIS fat when compared to sedentary obese animals (Kolahdouzi et al., 2019). Interestingly, this effect appears to be more pronounced with higher intensity exercise. For example, aerobic-interval training was more effective at improving vessel density in obese rats compared to lower intensity continuous training (Kolahdouzi et al., 2019). In agreement with these results, a recent clinical trial in men and women with insulin resistance found that both moderate intensity continuous training and sprint interval training improved vessel density in SUB adipose tissue (Honkala et al., 2020). Mechanistically, it has recently been suggested that murine double minute-2 (MDM2) may play a role in exercise-mediated increase in angiogenesis in WAT. *Mdm2* expression increases in both SUB and VIS fat in trained mice and knockdown of *Mdm2 in vitro* resulted in a decrease in capillary growth following adrenergic stimulation (Loustau et al., 2020). In comparison to WAT, less is known about the effects of exercise on angiogenesis in BAT. One study found that exercise in obese mice resulted in an increased expression of vascular endothelial growth factor A (VEGFA) and increased BAT mass relative to sedentary groups (Fu et al., 2021). In contrast, another study found that angiogenic gene expression increased in WAT, but was unchanged in BAT (Lee, 2018). While it is clear that exercise improves adipose tissue function, more work is needed to determine the role of increased angiogenesis in this observation, particularly in BAT.

### Fibrotic Remodeling

Obesity is known to cause increased fibrosis in adipose tissue which has been linked to metabolic dysfunction by limiting the healthy expansion of adipose tissue (Khan et al., 2009). Minimal work has been done investigating a direct link between exercise and fibrosis in adipose tissue. One study found that treadmill training in mice fed a HFD decreased fibrosis in VIS adipose tissue measured by decreased Picro Sirius red staining and decreased fibrotic gene expression (Kawanishi et al., 2013b). Additionally, another study investigating fibrosis in obese mice following exercise intervention found that exercise attenuated collagen deposition and fibrotic gene expression in VIS adipose tissue (Li et al., 2021). In comparison, aerobic exercise in obese adults did not affect any markers of fibrosis in SUB adipose tissue (Van Pelt et al., 2017). This discrepancy could be due to the fact that exercise in the human study was self-reported and utilized a different fat depot making it difficult to draw conclusions between species.

### Immune Remodeling

Infiltration of inflammatory immune cells in adipose tissue has be extensively studied and is known to contribute to obesity-related inflammation and insulin resistance (Liu and Nikolajczyk, 2019). Exercise is known to be immunomodulatory and thus has the potential to alleviate obesity-induced inflammation in adipose tissue. Macrophages are frequently implicated as a major contributor to adipose tissue inflammation and exercise has been shown to consistently reduce inflammatory macrophages in adipose tissue (Kawanishi et al., 2013a; Geng et al., 2019) and can shift the phenotype to an anti-inflammatory M2 macrophage (Kolahdouzi et al., 2019). In addition to a reduction in macrophage infiltration, exercise can reduce the amount of CD8^+^ T cell in adipose tissue with obesity (Kawanishi et al., 2013a). While we know that obesity leads to progressive low-grade inflammation associated with impaired insulin action in adipose tissue, even a shorter bout of exercise can reverse these effects. Thus, only 2 h of treadmill running in mice was able to cause noticeable anti-inflammatory effects in SUB fat of mice fed HFD (Macpherson et al., 2015). Mechanistically, acute exercise raised the expression of anti-inflammatory cytokines IL6 and IL10 and shifted the M1 macrophage phenotype toward the M2 phenotype in SUB fat of mice fed the HFD, which led to an insulin sensitizing effect. While this study associates exercise with better immune phenotype in adipose tissue, the causal mechanisms underlying these beneficial effects are still unknown.

Immune infiltrates in response to exercise have not been extensively studied in BAT when compared to WAT, however, one study found that exercise training in mice resulted in increased BAT mass and anti-inflammatory gene expression (Fu et al., 2021), suggesting the anti-inflammatory effects observed in WAT may also occur in BAT.

## Effects of Exercise on WAT Metabolic Function

There are well documented effects of exercise on mitochondrial activity (Stanford et al., 2015a,b), gene expression (Stanford et al., 2015a,b), and adipokine secretion (Bradley et al., 2008; Golbidi and Laher, 2014). Most notably, exercise increases peroxisome proliferator-activated receptor gamma coactivator 1-alpha (PGC-1a) expression in both WAT and BAT (Sutherland et al., 2009), increases UCP1 in WAT (Stanford et al., 2015a), and may increase plasma adiponectin levels (Lehnig and Stanford, 2018). Recent work has also shown that transplanting SUB fat from exercised mice to sedentary mice improves insulin sensitivity (Stanford et al., 2015a,b). The adaptations listed above are usually referred to as WAT beiging, which is specific to SUB depots in different locations. Emerging evidence suggests that the browning effect in WAT seen with exercise may be mediated, in part, by exercise-induced myokines and hormones such as IL-6, fibroblast growth factor 21 (FGF21), and Irisin (Severinsen et al., 2020). For example, FGF21 is known to stimulate WAT browning (Fisher et al., 2012) and acute exercise has been shown to increase circulating FGF21 in both mice and humans (Kim et al., 2013). The pro-browning effect of exercise has been mostly shown in rodents as humans seem refractory to this effect. Indeed, short-term (10 days) endurance training in young lean male subjects did not increase the mRNA expression of brown adipose tissue genes, mitochondrial content or fatty acid oxidation in SUB fat (Camera et al., 2010). Similarly, 6 weeks of endurance training did not increase brown and beige selective gene expression in abdominal or gluteofemoral SUB of obese subjects (Tsiloulis et al., 2018). These discrepant findings in humans and rodents could be due to differences between human and mice in terms of body size, cold tolerance and brown/beige content. The browning effect of exercise has been reviewed extensively and the reader is referred to recent reviews for more info on this topic (Sepa-Kishi and Ceddia, 2016; Townsend and Wright, 2019). Taken together, exercise intervention can improve both local WAT function and systemic metabolic health potentially through improvements in adipokine secretion.

## BAT Adaptations to Exercise

The effects of exercise on BAT are less clear in comparison to WAT adaptations. Considering that both exercise and BAT increase thermogenesis and energy expenditure, some have hypothesized that exercise may downregulate BAT to maintain body temperature (Lehnig and Stanford, 2018). One study found that treadmill exercise in rats resulted in a decrease in BAT mass, UCP1 expression, PGC-1a expression, and fatty acid oxidation (Wu et al., 2014). In human studies, endurance training has been linked to lower metabolic activity in BAT (Vosselman et al., 2015) and decreased insulin-stimulated glucose uptake following training (Motiani et al., 2017). Decreased BAT with exercise may seem counterintuitive to improvements in metabolic health, however, more work is needed to identify mechanisms of exercise-induced adaptions to BAT in both rodent models and humans to fully understand this relationship.

## Summary and Conclusion

Exercise improves adipose tissue cellularity, stimulates angiogenesis, and improves metabolic function of WAT (Figure 1). Also, exercise may limit fibrosis and pro-inflammatory immune cell infiltration in WAT. Moreover, exercise may improve the endocrine function of WAT by influencing the release of adipokines that attenuate systemic metabolic dysfunction, however, conflicting results have been observed. While many of the adaptations to WAT listed above may occur in BAT, less is known about the exercise-induced adaptations to BAT. While some have observed decreased BAT activity with exercise, it is clear this does not entirely prevent exercise-induced improvements in metabolic health. Taken together, exercise exerts positive changes to adipose tissue that promote healthier adipocytes and improve metabolic homeostasis. However, there are important questions that remain to be addressed: (1) how are stromal vascular cells within adipose tissue including APCs sensing exercise? (2) what are the systemic or local exercise-related signals that these cells are responding to and how is this response mediated at the cellular level? Does exercise impact proliferation/differentiation of APCs? What is the relative contribution of skeletal muscle versus BAT/beige adipose tissue to thermogenesis during exercise? Future studies are needed to address these topics which will help understand the mechanisms underlying the exercise effect on adipose tissue and metabolic health.

## Author Contributions

JG wrote the manuscript. SB critically revised the manuscript. Both authors approved the submitted version.

## Conflict of Interest

The authors declare that the research was conducted in the absence of any commercial or financial relationships that could be construed as a potential conflict of interest.

## Publisher’s Note

All claims expressed in this article are solely those of the authors and do not necessarily represent those of their affiliated organizations, or those of the publisher, the editors and the reviewers. Any product that may be evaluated in this article, or claim that may be made by its manufacturer, is not guaranteed or endorsed by the publisher.

## Abbreviations

BMI: body mass index
APC: adipose progenitor cell
SVF: stromal vascular fraction
SUB: subcutaneous
VIS: visceral
WAT: white adipose tissue
BAT: brown adipose tissue
UCP1: uncoupling protein 1
Mdm2: murine double minute-2
VEGFA: vascular endothelial growth factor A
HFD: high fat diet
FGF21: fibroblast growth factor 21
PGC-1a: peroxisome proliferator-activated receptor gamma coactivator 1-alpha.
